# Drivers of employment dynamics of AI innovators

**DOI:** 10.1016/j.techfore.2024.123249

**Published:** 2024-04

**Authors:** Giacomo Damioli, Vincent Van Roy, Dániel Vértesy, Marco Vivarelli

**Affiliations:** a European Commission, Joint Research Centre (JRC), Italy; b University of Strasbourg, France; c University of Bremen, Germany; d European Commission, Joint Research Centre (JRC), Spain; e KU Leuven, Belgium; f Idea Consult, Belgium; g International Telecommunication Union, Switzerland; h UNU-MERIT, the Netherlands; i Catholic University of Milano, Italy; j IZA, Germany

**Keywords:** Innovation, Technological change, Artificial intelligence, Patents, Employment, Job-creation

## Abstract

Based on an analysis of companies developing artificial intelligence (AI) technologies, this study offers fresh evidence on the role of innovation as one of the drivers of employment growth. GMM-SYS estimates on a worldwide longitudinal dataset covering 4,184 firms that patented inventions involving AI technologies between 2000 and 2016 show a positive and significant impact of AI patent families on employment. The effect, presumably of product innovations, is small in magnitude and limited to service sectors and younger firms, which are at the forefront of the leaders of the AI revolution. We also detect some evidence of increasing returns, suggesting that innovative companies more focused on AI technologies are achieving larger impacts in terms of job creation.

## Highlights

• This paper studies the possible job creation effect of AI innovation activity.
• GMM-SYS estimates show that patenting in AI increases employment of AI innovators.
• Job creation is limited to service sectors and younger firms.
• Specialization in AI inventions increases employment returns of AI patents.
• The findings show a labor-friendly nature of AI innovative activity.

## 1 Introduction

The past two decades have witnessed major developments in artificial intelligence (AI) technologies. As with previous technological revolutions, such as the diffusion of ICTs in the last decades of the past century, artificial intelligence displays remarkable and pervasive impacts across firms, industries, economies and societies. While in this work we will focus on the drivers of employment evolution within AI innovators, the current debate is mainly focused on the potential disruptive effect of automation in adopting firms.

In particular, the possible adverse impact of AI diffusion and robotics on employment has generated concern and lively discussion in academic debate and in society as a whole. The fear of ‘technological unemployment’ has resurfaced with the arrival of the internet of things, self-driving cars and a general widespread use of AI applications and robots (see Brynjolfsson and McAfee, 2011, Brynjolfsson and McAfee, 2014). AI, self-learning algorithms and human-imitating robots can perform tasks usually requiring human beings' intelligence and dexterity (such as speech recognition, decision-making advice, disease diagnostics, translation of complex documents, performance of unhealthy and dangerous tasks and so forth; see Frey and Osborne, 2017; Ernst et al., 2018). Indeed, Dobbs et al. (2015) of the McKinsey Global Institute estimate that compared with the industrial revolution of the 19th century, automation and disruption of society by AI are happening 10 times faster and at 300 times the scale.

However, current debate and the extant literature (as discussed in the following section) have dealt almost exclusively with the demand side, by looking at the potential labour-saving effect that may occur among users of AI and robotics technologies conceived as process innovations in downstream sectors. An obvious gap in the literature exists regarding a possible job-creation effect in the supply side among developers of AI and robotics technologies conceived as product innovations in upstream sectors. Indeed, according to the Schumpeterian literature (see Schumpeter, 1912; Porto et al., 2021), technological change entails both labour-saving process innovation and product innovation. The introduction of new products (both in manufacturing and services) can give rise to new branches of production and create additional employment opportunities.^1^

As was the case for ICT, AI and robots can be seen simultaneously as labour-saving process innovations in user sectors (e.g. the massive adoption of robots in the automotive industry) and as labour-friendly product innovations in the supply industries (e.g. the electronic and machinery industries producing robots, or the scientific and technical services where AI algorithms are conceived).^2^ In this framework, new AI products not only entail a substantial expansion of existing sectors (such as those related to ICT, software and robotisation), but also the creation of brand-new employment opportunities, such as those related to data processing, transactional procedures, customerisation, remote collaboration, etc. Very few studies have addressed this side of the coin (the “bright side”), while most previous literature has focused on the “dark side” of the coin, i.e. the labour-saving effect of automation technologies in the user industries. Specifically, at present and to the best of our knowledge, Alderucci et al. (2020), Yang (2022) and ourselves (Damioli et al., 2022) are the only scholars who have addressed the possible labour-friendly effect of AI in the upstream sectors (see the next section for discussion of these works).

The aim of this study is to assess the possible job-creation impact of AI technologies on the supply side, i.e. among the developers and providers of the new knowledge base. As our research purpose is to investigate the labour-friendly nature of AI technologies, our empirical sample does not aim to be representative but is deliberately limited to those companies that are active in AI and robotic patenting. In more detail, our analysis is based on a worldwide set of 4,184 front-runner companies that patented relevant technologies over the 2000–2016 time span. Controlling for the other main drivers of employment at the firm level, namely output, cost of labour, capital formation and innovation other than AI, we characterise the labour-friendly impact of AI and robots initially detected in Damioli et al. (2022)^3^ by investigating the heterogeneous impact of AI technologies on employment across manufacturing and service sectors and age categories of firms. Moreover, we assess to what extent the impact on employment differs for AI-specialized firms, i.e. those having a relatively higher share of AI patents in their technological basis. The construction of a wider database^4^ and the investigation of heterogeneous patterns among AI innovators are the main novelties of this study in comparison with the previous (scant) literature focusing on the supply side (see also next section).

The article is organised as follows. Section 2 summarises the extant literature, emphasising its limited focus on the labour-saving impact detectable in the adoption of new technologies, and further highlights the purpose and novelty of this study. Section 3 describes the microeconometric methodology used in our analysis. Section 4 discusses the data and the sample used for the empirical analysis. Section 5 presents and discusses the main results. Finally, Section 6 wraps up and puts forward some conclusions and tentative policy implications.

## 2 The literature

As mentioned above, the extant economic literature mainly focuses on the possible labour-saving effect of AI and robots, conceived as process innovation in the user industries. Thus, recent contributions form part of an established tradition of studies devoted to the controversial relationship between technology and employment (for a long-term historical analysis, see Staccioli and Virgillito, 2021; for a recent theoretical reprise of the issue, see Acemoglu and Restrepo, 2018, Acemoglu and Restrepo, 2019).^5^

As far as the employment consequences of the current widespread diffusion of AI and robots are concerned, the empirical literature provides both macroeconomic forecasting scenarios and some sectoral and microeconomic evidence. As far as the macro scenarios are concerned, Frey and Osborne (2017), using a Gaussian process classifier applied to data from O*Net and the US Department of Labor, predict that 47% of occupational categories, mostly middle- and low-skilled professions (including a wide range of service/white-collar/cognitive tasks such as accountancy, health professions, logistics, legal work, translation and technical writing) are at a high risk of being substituted by AI algorithms and robots.

However, Arntz et al., 2016, Arntz et al., 2017, proposing a similar, but more fine-grained approach that takes into consideration the heterogeneity of workers' tasks, conclude that only 9% of US jobs are at potential risk of automation. Their main message is that, within the same occupation, some tasks can be automatized while others cannot and therefore the associated job can be preserved.

Extending the analysis to a multi-country approach, Nedelkoska and Quintini (2018) estimate the risk of automation for individual jobs in 32 OECD countries. Their evidence shows that about 14% of jobs are highly automatable (probability of automation over 70%), while another 32% of jobs present a 50 to 70% risk of being substituted, pointing to the possibility of significant changes in the way these jobs will be carried out as a result of automation.

At the European level, Pouliakasn (2018), using data on tasks and skill needs collected by the European Skills and Jobs Survey (ESJS), bundles jobs according to their estimated risk of automation. Following Frey and Osborne (2017) and Nedelkoska and Quintini (2018), the author utilises highly disaggregated job descriptions and shows that 14% of EU adult workers face a very high risk of automation.

Turning our attention to the sectoral and microeconomic evidence, the extant empirical literature has focused particularly on robotisation within adopting firms. For instance, Acemoglu and Restrepo (2020) investigate the employment effect of exposure to robots, using the sectoral “International Federation of Robotics” (IFR) data (national penetration rates instrumented by European data). According to their 2SLS estimates, robotisation had a significant negative impact on the change in employment and wages in each US local labour market over the period 1990–2007. More specifically, they show that one more robot per thousand workers reduces the employment/population ratio by about 0.18-0.34%.^6^

Graetz and Michaels (2018) use panel data on robot adoption (IFR and EUKLEMS data to estimate robot density) within industries in 17 countries from 1993 to 2007: their main finding is at odds with the previous study, since they conclude that robots do not significantly reduce total employment, although they do reduce the low-skilled workers' employment share.

Finally, Dauth et al. (2021) propose an empirical exercise for Germany using IFR data over the 1994–2014 time span, adopting a measure of local robot exposure for each region. They find that although industrial robots imply job losses in the manufacturing sector, employment in the non-manufacturing sectors increases and, overall, counterbalances the negative impact in manufacturing.

As is clear from the discussion above, the existing literature is mainly concerned with the possibly negative employment impact of process innovations induced by AI technologies and robots, while there is a lack of focus on the product innovation aspects of these technologies, which possibly exert labour-friendly effects in the upstream sectors. This is surprising, because both theory (see Katsoulacos, 1984; Vivarelli, 1995; Edquist et al., 2001) and empirical evidence (see Freeman and Soete, 1987, Freeman and Soete, 1994; Bogliacino and Pianta, 2010; Bogliacino et al., 2012; Van Roy et al., 2018) indicate that product innovations are key drivers of new job creation.

However, the labour-friendly impact of product innovations may vary according to their nature. Indeed, new products may be either brand-new entities or substitutes of obsolete ones. If revenues from new products cannibalise the sales of old ones, the net result in terms of employment expansion might be ambiguous. In other words the “welfare effect” should be compared with the “substitution effect” (using the terminology originally put forward by Katsoulacos, 1984, Katsoulacos, 1986; see also Vivarelli, 1995 and Dosi et al., 2021). Empirically, this means that the expected sign of the correlation between product innovation and employment is positive, but uncertain in significance and magnitude.

Moreover, we have to underline and investigate the considerable *heterogeneity* among AI innovators. As is the case for any kind of innovation, the current AI revolution comprises leaders and followers (see Schumpeter, 1912), emerging sectors and mature ones (see Freeman and Soete, 1987), radical innovations and incremental ones (see Dosi, 1988), in a continuous overlapping process of novelty creation and diffusion. If we take into account the variety among innovators (see Dosi, 2023), it may well be the case that a possibly labour-friendly impact of AI innovation is not equally spread among the AI innovators, but rather is concentrated in services (those inventing and producing the AI algorithms) and in the front-runner companies in patenting AI and robot technologies worldwide, such as relatively young companies^7^ and firms devoting their innovative activity mostly to AI technologies (pointing to a kind of “increasing returns” in the employment multiplier of AI technologies).

These hypotheses match what has been proposed in the recent literature, showing that AI can be considered a key pervasive technology driving a change in the technological paradigm (see WIPO, 2019; Petralia, 2020a; Damioli et al., 2021; Santarelli et al., 2023). As was the case in former technological revolutions (e.g. the automobile driving the Fordist paradigm or the PC and internet driving that of ICT, see Dosi, 1988; Petralia, 2020b), the expectation is that new AI products (such as algorithms) will be massively used by virtually all the downstream economic sectors, so spurring a labour-friendly effect among the main providers of these new products, which are indeed concentrated in services, young companies and AI-intensive firms.

Bearing in mind the discussion above, our theoretical expectation is that the “welfare effect” would be dominant (given the novelty of the AI products) and that the job creation impact would be mostly detected in the leading AI companies, given the pervasiveness of their AI products and the corresponding exponential increase in demand. Indeed, the (scant) extant empirical literature dealing with the possible labor-friendly nature of AI technologies in the upstream sectors (those patenting and providing the new technologies) supports the prevalence of a “welfare effect”, and so an overall positive impact of AI technologies on employment at the firm level.

In more detail, Alderucci et al. (2020) put forward a machine learning methodology to identify AI related granted patents from the USPTO patent corpus over the period 1990–2018 and then match them with firm-level microdata generated by the US Census. Among their various empirical tests, the authors run a difference-in-differences specification centered on the timing of the first AI-related patent by a given company. They find a positive and significant employment impact of the AI treatment (a 0/1 dummy), growing over time (Alderucci et al., 2020, Table 7).

Yang (2022) extracts AI-related patents from the Taiwan Patent Office, using a keyword-matching approach similar to that proposed by Van Roy et al. (2020), and also used in this study. Then, the author matches these data with a firm-level longitudinal dataset of Taiwanese electronics firms and investigates the impact of AI technologies on both productivity and employment. As far as the latter is concerned, a positive and significant effect is detected, using both AI treatment and number of AI-related patents; however, this effect fades when employment growth (instead of employment) level is used as the dependent variable (Yang, 2022, Table 6).

Finally, in a letter article which can be seen as an antecedent of this study, Damioli et al. (2022) construct a novel worldwide longitudinal database, obtained by merging the EPO PATSTAT and BvD-ORBIS sources, and identifying more than3,500 companies that patented AI-related inventions over the period 2000–2016 (3,510 firms for a total of 26,137 observations). Their main result reveals a significant labour-friendly impact of AI patenting (Damioli et al., 2022, Table 1).

**Table 1:** Summary statistics of the dependent and explanatory variables in the full sample.

Variable name	Mean	SD	Min	Max
Employment	5,161	23,328	1	552,810
Turnover	1.46E+09	7.84E+09	10,000	3.48E+11
Gross investments	22.5	78.3	−97.8	894.1
Cost of labour per employee	34,586	32,050	2.3	422,000
AI patent families	0.3	1.6	0	78
Non-AI patent families	32.4	148.4	0	6,601
AI patent family size	0.3	1.0	0	51.5
Non-AI patent family size	1.5	2.2	0	44.5

*Notes*: the full sample includes 28,840 observations and 4,184 firms. Employment is the number of employees. Turnover, cost of labour per employee, and fixed assets are expressed in EURs. Gross investments are shown as yearly percentage changes.

Building on the latter study, the main novelties of the present work are the following. Firstly, we further expand our database^8^, extending the coverage of automation in our keywords algorithm (see Table A1 in the Appendix) and reaching 4,184 firms for a total of 28,840 available observations. Secondly, and consistently with the discussion put forward above, we investigate in detail the *heterogeneity* among the AI innovators, to detect whether the labour-friendly impact of new technologies is equally spread or rather concentrated in particular categories of firms, e.g. younger companies, those operating in service industries or those leading the process of provision of AI innovations.

**Fig. 1:**
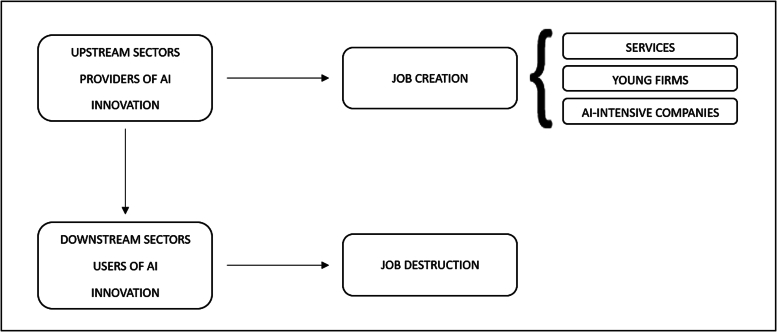
Conceptual framework.

In the following Fig. 1, we summarize the arguments discussed in this paragraph.

## 3 Econometric methodology

We use a stochastic labour demand model derived from a Cobb-Douglas utility function to investigate the potential impact of AI innovations on employment. In particular, we extend a standard labour demand function with a technology factor (i.e. a proxy for innovation) to control for the effect of technological change on employment. This specification has been widely used in prior literature for longitudinal firm-level analysis (see Van Reenen, 1997; Lachenmaier and Rottmann, 2011; Bogliacino et al., 2012; Van Roy et al., 2018). Along these lines, the labour demand function for a panel of firms *i* over time *t* is defined as:

(1) $$\begin{array}{l} l_{i,t}=\beta_{1}y_{i,t}+\beta_{2}w_{i,t}+\beta_{3}I_{i,t}+\beta_{4}\;{\mathrm{innov}}_{i,t}+\mu_{i}+\varepsilon_{i,t}\\ \text{with}:i=1,..,n;t=1,..,T \end{array}$$

Lower case letters denote natural logarithms, *l* corresponds to labour (proxied by employment level), *y* to output (proxied by turnover), *w* to wages (proxied by labour cost per employee), and *I* to gross investments (proxied by growth in fixed capital). The labour demand function is augmented with a variable *innov* capturing technological change due to innovations. Lastly, μ is an unobserved firm-specific and time-invariant effect and ε the usual error term.

We subsequently move from this static expression (1) to a dynamic specification as given in (2), in order to account for viscosity in labour demand (see Arellano and Bond, 1991; Van Reenen, 1997):

(2) $$\begin{array}{l} l_{i,t}={\mathrm{\alpha l}}_{i,t‐1}+\beta_{1}y_{i,t}+\beta_{2}w_{i,t}+\beta_{3}I_{i,t}+\beta_{4}\;{\mathrm{innov}}_{i,t}+\mu_{i}+\varepsilon_{i,t}\\ \mathrm{with}:i=1,..,n;t=1,..,T \end{array}$$

As the measure for technological change we use AI and non-AI patent families (respectively denoted by $\mathrm{Pa}t_{i,t}^{\mathrm{AI}}$ and $\mathrm{Pa}t_{i,t}^{\mathrm{Non}-\mathrm{AI}}$), as outlined in specification (3). In essence, this specification aims to proxy for the technological progress brought about by firms through the development of innovative and marketable (and hence patentable) technologies in AI and non-AI related fields:

(3) $$\begin{array}{l} \beta_{4}{\mathrm{innov}}_{i,t}={\mathrm{\gamma Pat}}_{i,t}^{\mathrm{AI}}+{\mathrm{\delta Pat}}_{i,t}^{\mathrm{Non}‐\mathrm{AI}}\\ \mathrm{with}:i=1,..,n;t=1,..,T \end{array}$$

Problems of simultaneity and endogeneity may occur in the dynamic labour demand Eq. (2) which could lead to biased estimations of the covariates.^9^ To remove this potential bias, we employ a system GMM estimation model as proposed by Blundell and Bond, 1998, Blundell and Bond, 2000. The system GMM approach uses instrumental variables to provide consistent and efficient estimates when dealing with dynamic panel data, as is the case for our data. In a system of equations (i.e. in level equations and equations in differences), lagged and differenced lagged variables are used to solve issues of persistency in times series and endogeneity.

Unfortunately, the lagged dependent variable may not be the only one to suffer from endogeneity. Other explanatory variables of the labour demand function may also be affected, as pointed out in prior literature (e.g. Bogliacino et al., 2012; Van Roy et al., 2018). It might be the case that wage and employment are simultaneously decided, while the output and investment decisions may be jointly affected by a temporary shock. Therefore, in line with previous studies, all the explanatory variables have been considered as potentially endogenous to labour demand and instrumented when needed.^10^

In more detail, in the level equation we used differenced values of the explanatory variables as instruments, i.e. twice- or thrice-lagged differences in labour demand, AI and non-AI patent families, gross investments and cost of labour. The level equations also include a set of sector, country and year dummies. In the equations in differences we employed twice- or thrice-lagged values of the above-mentioned right-hand side variables as instruments. To define the lag limits of instruments we follow Roodman, 2009a, Roodman, 2009b advice of minimising the number of instruments and setting them so as to satisfy the autocorrelation tests.^11^

## 4 Data and sample

### 4.1 Data

Our novel dataset is based on a worldwide panel of firms patenting in AI. While traditionally considered as a proper measure of a commercially valuable innovation output (Griliches, 1990; Ernst, 1995), the limitations of patents in capturing innovations are well known (e.g. Hussinger, 2006; Hall et al., 2014): for instance, the fact that companies may prefer not to patent their inventions to keep them secret. Moreover, patents better proxy product rather than process innovations, which are often embodied in machineries and can be more easily kept secret than products (Levin et al., 1987; Lissoni et al., 2013). However, patents have the appealing advantage of allowing identification on a global scale of firms that innovate in AI. Accordingly, an increasing number of studies rely on patents to track and analyse the development and adoption of AI technologies in production processes and their economic consequences (Webb et al., 2018; WIPO, 2019; Baruffaldi et al., 2020; Van Roy et al., 2020; Damioli et al., 2021; Martinelli et al., 2021).

However, the identification of patents related to AI technologies is a challenging task. There is no established definition of the boundaries of the AI technological domain, nor an agreed methodology for empirically singling it out. On the one hand, conceptual definitions of AI typically insist on the ability of a system to perform human-like cognitive functions (learning, understanding, reasoning and interacting) with the aim of obtaining rational outcomes (Ertel, 2018; Russell and Norvig, 2016). On the other hand, albeit AI technologies focus on a core of software technologies including, inter alia, machine learning, neural networks, logic programming and speech recognition, various studies consider a broader definition of AI a combination of software and hardware components, as well as functional applications such as robots and “big data” (European Commission, 2018; Fujii and Managi, 2018; WIPO, 2019).

Van Roy et al. (2020), on which this study relies upon for the selection of patents, used a keyword-based search of AI-related terms in the title or the abstract of patents. An analogous approach to select AI patents has been pursued in previous studies on AI and robotics technologies (Keisner et al., 2015; De Prato et al., 2019; European Commission, 2018; Cockburn et al., 2019; WIPO, 2019; Baruffaldi et al., 2020). Some of these studies applied their keywords' search to patents falling in pre-selected technological classes (Keisner et al., 2015; Cockburn et al., 2019; WIPO, 2019), while others relied on all patents falling in specific technological classes mapping to AI technology areas (e.g. Inaba and Squicciarini, 2017; Fujii and Managi, 2018; OECD, 2017).^12^

In this study, we rather prefer unrestricting our patents to pre-determined technological classes in reason of the transversal nature of AI technologies that, as any other general-purpose technology, cut through many scientific disciplines and technological domains (Bianchini et al., 2022; WIPO, 2019). Our list of keywords takes stock of the findings of prior relevant literature and is shown in Table A1 in Appendix A.

**Fig. A1:**
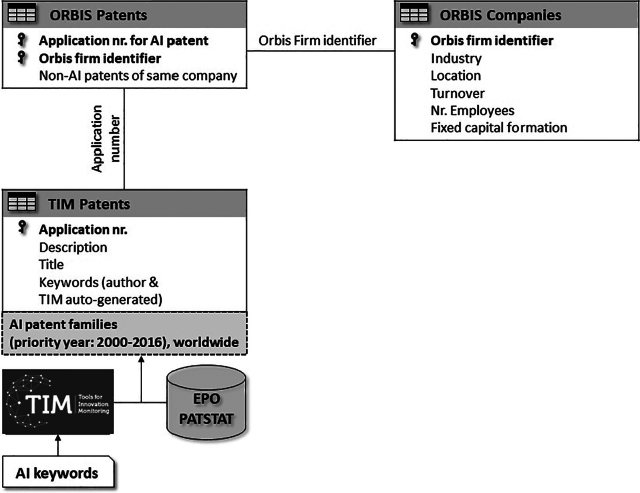
Data matching procedure.

The Spring 2018 edition of the PATSTAT database of the European Patent office has been screened with text-mining tools to extract all patent families that contain any of the relevant AI-related keywords in their title or abstract (see Van Roy et al., 2020 for more information on the methodology). The use of patent families prevents double counting of similar inventions filed in different patent offices and countries. Subsequently, we retrieved key firm-level accounting information for AI patent applicants from Bureau van Dijk Electronic Publishing (BvD) ORBIS databases. While the issues of coverage and data availability are known limitations of ORBIS, these are by far outweighed by its advantage of offering a comprehensive cross-country micro-level dataset for scientific research purposes (e.g. Gal, 2013; Hallak and Harasztosi, 2019). We used patent application numbers to track applicant firms in the ORBIS Intellectual Property database, which we also exploited to retrieve all other non-AI patent applications of such firms, and matched location and economic information from the ORBIS Companies database. Fig. A1 in Appendix A shows a synopsis chart illustrating the data collection process.

### 4.2 Variables and sample

We cleaned the firm-level accounting information of outliers in both level and growth rates and missing data for the variables on employment, value added, fixed assets and cost of labour.^13^ This resulted in a sample of 4,184 AI-patenting firms with 28,840 observations over the years 2000–2016. This database covers a global set of firms from both the manufacturing and service sectors. It comprises information on firms' patenting activities in AI and non-AI related fields, accounting information (including employment, turnover, value added, capital formation, and cost of labour), year of birth or consolidation, country location, and industrial activity (NACE sector at 2-digit level).

We use the natural logarithm of the number of employees as the dependent variable of the labour demand function.^14^ In line with Eq. (2), independent variables include the natural logarithm of firm turnover, labour cost per employee and gross investments proxied by the annual growth in fixed assets. Following prior studies, both turnover and gross investments are expected to have a positive impact on labour demand, while higher labour costs may decrease employment levels (e.g. Bogliacino et al., 2012; Van Roy et al., 2018). We also include various dummies in the models to capture industry-, time- and country-specific factors that could influence labour dynamics.

The natural logarithm of the number of AI patents constitutes the explanatory variable of interest, which permits measurement of a firm's AI technological basis. Moreover, we also include a firm's innovative efforts in non-AI related fields with the number of non-AI patents in a natural logarithm. As such, we avoid overestimating the impact of AI technologies on employment.

As its economic or technological value may vary widely from patent to patent, the use of patent counts may fall short in capturing quality differences across firms' technologies. To this purpose, a wide range of patent quality indicators have been proposed in the literature, including patent renewals, patent family sizes, and back- and forward citations (Harhoff et al., 2003; Hall et al., 2005; Gambardella et al., 2008; Neuhäusler et al., 2011; Squicciarini et al., 2013; Van Roy et al., 2018). Although forward citations are among the most widely used indicators to measure patent quality, they are difficult to apply in our analysis, as AI patenting is a recent phenomenon, experiencing a sharp increase since 2015 (Cockburn et al., 2019; WIPO, 2019; Van Roy et al., 2020). For this reason, we use patent family size, proxied by the number of patent offices at which a given invention has been protected, as an alternative measure of patent quality. Patents covering different jurisdictions and a larger geographical scope of protection are found to be more valuable, as applicants tend to take on the higher associated requirements in terms of time, effort and cost of filing patents abroad only for those that are worth it (Harhoff et al., 2003; Lanjouw and Schankerman, 2004; Johnstone et al., 2012). To obtain a measure at the firm level, we took the family size of the patent application as the average size of all applications a company made in each year, and we computed it separately for AI and non-AI patents. Before log transformation, all variables were shifted positively by 1 in order to accommodate 0 values.

Table 1 reports the summary statistics of the dependent and explanatory variables used in the estimations. Firms in the sample report an average of over five thousand employees, due to the significant presence of big corporations. AI patent families are relatively low, about one AI-related family per year for every three firms in the sample, due to a highly skewed distribution with a large number of firm-year pairs without any AI families (about 86% of all observations). By contrast, non-AI patent activity is considerable, at 32 yearly patent families on average per firm, which confirms that the sample includes highly innovative companies. As for patent family size, non-AI patents are filed in more patent offices than AI applications, even after conditioning on the firm-year pairs with at least one application: the conditioned average of the family size is 1.9 for AI patents and 2.5 for non-AI patents.

Table 2 shows the distribution of firms according to their main activity, age and AI intensity, these being the subsamples of particular interest for the following analyses.^15^ The majority of the firms in the sample are active in manufacturing (57%, vs. 43% in services) and have been founded or consolidated since 1990 (66%). The largest share of firms belongs to electronics (23%) and machinery (15%) within manufacturing industries, and telecommunications (17%) and scientific services within service industries (10%). Table B2 in Appendix B provides a more detailed distribution of firms across sectors. We also consider intensity in AI patenting as measured by the ratio of AI patents over the total number of patents in the period. In particular, we consider AI-specialized companies those with a ratio of AI patents over total patents above the revealed median in our sample (5%).^16^

**Table 2:** Distribution of firms across sectors, age, AI intensity and firm size.

	Full sample			
	Observations		Firms	
	Number	Perc.	Number	Perc.
Sector				
Services	11,713	40.61	1,810	43.26
Manufacturing	17,127	59.39	2,374	56.74
Age of firm				
Founded before 1990	11,077	38.41	1,414	33.80
Founded after 1990	17,763	61.59	2,770	66.20
AI intensity				
AI-specialized	14,327	49.68	2,246	53.68
Non-AI-specialized	14,513	50.32	1,938	46.32
Total	28,840	100.00	4,184	100.00

*Notes:* Age is based on the year of foundation or consolidation of the firm. AI-specialized companies are those with a share of AI patents over total patents in the period which is above the median.

In terms of geographical distribution, the majority of firms are located in Asia (61%). This large percentage is driven by the dominating AI patenting activity of Japanese and South Korean firms, as highlighted in prior studies (WIPO, 2019; Van Roy et al., 2020). About 32% of the firms are located in Europe, with highest percentages in Germany, France, Italy and the United Kingdom. Lastly, firms in the United States constitute around 6% of the sample.^17^

## 5 Econometric results

### 5.1 Model selection

To support the chosen methodology, Table C1 in Appendix C reports the estimation coefficients for pooled ordinary least square (POLS), fixed-effects (FE) and system generalized method of moments (SYS-GMM) models. Lagged employment is highly significant in all three of the different estimations tested. Its magnitude ranges from 0.43 in the FE estimation, to 0.82 in the POLS estimations. While FE tends to underestimate the impact of the lagged dependent variable, POLS, by contrast, overestimates it. Solving for persistency and endogeneity, it is therefore to be expected that the SYS-GMM estimates for the lagged dependent variable fall within these two boundaries. In fact, the coefficient for lagged employment, obtained from the one-step SYS-GMM at 0.45, meets the above methodological expectation. This finding applies to the standard setup as well as to an alternative one where, in order to reflect patent quality, family size is used instead of patent counts (with FE estimates for lagged employment at 0.44, SYS-GMM at 0.45 and POLS at 0.83). Unsurprisingly, labour demand is persistent and autoregressive, confirming its path dependency.

As per diagnostics for the baseline SYS-GMM model, the Wald test on the overall significance of the regressions and the LM tests on AR(1), AR(2) and AR(3) autocorrelation dynamics confirm the robustness of the model (requiring thrice-lagged instrumentation in both the baseline regression and in some of the splits, twice-lagged instrumentation in the remaining cases). Evidence from the Hansen test shows that the null of adequate instruments is rejected. This is not surprising, as it is well known that the Hansen test over-rejects the null in very large samples (Blundell and Bond, 2000; Roodman, 2009a). Following the extant literature, we estimated the same model and computed the Hansen test in different random sub-samples comprising 10% of the original observations: in all models the null of the Hansen test was not rejected, which is reassuring as regards instrument validity.^18^ Finally, given the observation that a high number of instruments may imply a downward bias in the standard errors for two-step SYS-GMM models (Roodman, 2009b), we opted for the more conservative one-step methodology.

### 5.2 Baseline results

The baseline estimation reported in Table 3 (applying the one-step SYS-GMM estimation on the full sample of 28,840 observations from 4,184 firms active in AI patenting) provides results that are in line with prior studies with a similar setup (Bogliacino et al., 2012; Van Roy et al., 2018; Pellegrino et al., 2019). The coefficients of the explanatory variables are significant and have the expected sign. We detect highly significant and large positive effects of lagged employment (0.45), which confirms the persistence of labour demand and turnover (0.37); a small and barely significant positive effect of gross investments (0.03)^19^; and a strong negative effect of the labour cost per employee (−0.48).

**Table 3:** Results from GMM-SYS analysis: baseline estimations and estimations split by industry and firm age.

	Baseline		Industry				Age of firm			
			Services		Manufacturing		Founded before 1990		Founded after 1990	
Employment t-1	0.447***	0.452***	0.482***	0.492***	0.415***	0.420***	0.270***	0.275***	0.473***	0.478***
	(0.000)	(0.000)	(0.000)	(0.000)	(0.000)	(0.000)	(0.000)	(0.000)	(0.000)	(0.000)
Turnover	0.368***	0.372***	0.305***	0.313***	0.307***	0.304***	0.491***	0.481***	0.292***	0.305***
	(0.000)	(0.000)	(0.000)	(0.000)	(0.000)	(0.000)	(0.000)	(0.000)	(0.000)	(0.000)
Gross investments	0.033*	0.033*	0.027*	0.027*	0.035	0.036	0.091	0.093	0.023*	0.023*
	(0.092)	(0.092)	(0.058)	(0.053)	(0.304)	(0.293)	(0.148)	(0.136)	(0.083)	(0.085)
Labour cost per employee	−0.475***	−0.481***	−0.412***	−0.422***	−0.515***	−0.521***	−0.591***	−0.606***	−0.430***	−0.433***
	(0.000)	(0.000)	(0.000)	(0.000)	(0.000)	(0.000)	(0.000)	(0.000)	(0.000)	(0.000)
AI patent families	0.023**		0.033*		0.012		0.009		0.032**	
	(0.042)		(0.093)		(0.421)		(0.610)		(0.031)	
Non-AI patent families	0.022***		0.045***		0.003		0.009		0.033***	
	(0.010)		(0.001)		(0.797)		(0.582)		(0.002)	
AI patent family size		0.016*		0.033**		0.004		0.000		0.026**
		(0.051)		(0.011)		(0.726)		(0.994)		(0.015)
Non-AI patent family size		0.008		0.027**		−0.010		0.005		0.009
		(0.356)		(0.043)		(0.299)		(0.751)		(0.383)
Wald test	6,944***	2.41E+11***	14,010***	4.59E+10***	1.02E+06***	168,677***	664.6***	1.19E+09***	3.87E+10***	638,108***
	(0.000)	(0.000)	(0.000)	(0.000)	(0.000)	(0.000)	(0.000)	(0.000)	(0.000)	(0.000)
Hansen test	147,971***	46,428***	58.58***	2.42E+09***	1.97E+09***	13,956***	709.9***	1.42E+21***	1.35E+21***	1.75E+23***
	(0.000)	(0.000)	(1.70E-08)	(0.000)	(0.000)	(0.000)	(0.000)	(0.000)	(0.000)	(0.000)
AR (1)	−12.02***	−11.90***	−10.10***	−10.08***	−7.800***	−7.734***	−4.588***	−4.592***	−12.17***	−12.03***
	(0.000)	(0.000)	(0.000)	(0.000)	(0.000)	(0.000)	(4.48E-06)	(4.39E-06)	(0.000)	(0.000)
AR (2)	−2.134**	−2.252**	−0.628	−0.676	−2.120**	−2.212**	−2.041**	−2.070**	−0.662	−0.733
	(0.033)	(0.024)	(0.530)	(0.499)	(0.034)	(0.027)	(0.041)	(0.038)	(0.508)	(0.464)
AR (3)	−0.634	−0.519			−0.285	−0.202	−1.485	−1.401		
	(0.526)	(0.604)			(0.775)	(0.840)	(0.138)	(0.161)		
Instruments	108	108	84	84	94	94	101	101	98	98
Obs.	28,840	28,840	11,713	11,713	17,127	17,127	11,077	11,077	17,763	17,763
N. of firms	4,184	4,184	1,810	1,810	2,374	2,374	1,414	1,414	2,770	2,770

*Notes:* All variables are taken in natural logs, apart from gross investments, which are expressed as the log difference of fixed assets between time *t* and *t-1*. All models include industry, country and year dummies. p-values derived from one-step GMM robust standard errors are reported in parentheses. Instrumental variables compromise 2- and 3-year lags. *** p < 0.01, ** p < 0.05, * p < 0.1.

Estimates of parameters associated with the focal variables of interest are positive and statistically significant and indicate an elasticity of labour demand to AI and non-AI patent families ranging from 2.2% to 2.3%. This finding is in line with the GMM-SYS outcome reported by Yang (2022, Table 8) for Taiwan and supports the employment-friendly nature of product innovation. However, when measures of patent quality are used, only AI patent families provide a (barely) significant positive effect on employment, while the effect of non-AI patents does not reach customary levels of statistical significance. Since results from weighted patents should be considered as more reliable, failing to find strong support for the expected labour-friendly nature of AI and non-AI innovation opens the way to a more granular investigation, to discover whether this (rather disappointing) outcome may be due to a composition effect, with some categories of companies benefiting from the job creation generated by new technologies while others do not.

### 5.3 Sample splits

In order to enrich our understanding of the impact of AI innovation on employment, results for three sample splits are presented in Table 3, Table 4, namely sector of main economic activity, age and AI intensity as defined in Section 4.2. We note that the overlaps between the groups singled out in the three splits are sufficiently limited to allow differentiated findings.^20^

**Table 4:** Results from GMM-SYS analysis: baseline estimations and estimations split by AI intensity.

	Baseline		AI intensity			
			AI specialized		Non-AI-specialized	
Employment t-1	0.447***	0.452***	0.544***	0.547***	0.343***	0.344***
	(0.000)	(0.000)	(0.000)	(0.000)	(0.000)	(0.000)
Turnover	0.368***	0.372***	0.263***	0.267***	0.432***	0.435***
	(0.000)	(0.000)	(0.000)	(0.000)	(0.000)	(0.000)
Gross investments	0.033*	0.033*	0.028**	0.027**	0.052	0.054
	(0.092)	(0.092)	(0.013)	(0.016)	(0.229)	(0.220)
Labour cost per employee	−0.475***	−0.481***	−0.471***	−0.472***	−0.472***	−0.478***
	(0.000)	(0.000)	(0.000)	(0.000)	(0.000)	(0.000)
AI patent families	0.023**		0.032**		0.016	
	(0.042)		(0.031)		(0.449)	
Non-AI patent families	0.022***		0.037***		0.003	
	(0.010)		(0.000)		(0.803)	
AI patent family size		0.016*		0.025*		0.005
		(0.051)		(0.061)		(0.678)
Non-AI patent family size		0.008		0.028***		−0.015
		(0.356)		(0.003)		(0.298)
Wald test	6,944***	2.41E+11***	2.29E+10***	1,596***	5.47E+11***	4.24E+06***
	(0.000)	(0.000)	(0.000)	(0.000)	(0.000)	(0.000)
Hansen test	147,971***	46,428***	4,954***	5,049***	8.81E+21***	3,150***
	(0.000)	(0.000)	(0.000)	(0.000)	(0.000)	(0.000)
AR (1)	−12.02***	−11.90***	−10.92***	−10.80***	−7.663***	−7.576***
	(0.000)	(0.000)	(0.000)	(0.000)	(0.000)	(0.000)
AR (2)	−2.134**	−2.252**	−2.053**	−2.115**	−1.727*	−1.762*
	(0.033)	(0.024)	(0.040)	(0.034)	(0.084)	(0.078)
AR (3)	−0.634	−0.519	−0.0961	−0.139	−0.152	−0.112
	(0.526)	(0.604)	(0.923)	(0.890)	(0.879)	(0.911)
Instruments	108	108	101	101	103	103
Obs.	28,840	28,840	14,327	14,327	14,513	14,513
N. of firms	4,184	4,184	2,246	2,246	1,938	1,938

*Notes:* All variables are taken in natural logs, apart from gross investments, which are expressed as the log difference of fixed assets between time *t* and *t-1*. All models include industry, country and year dummies. p-values derived from one-step GMM robust standard errors are reported in parentheses. Instrumental variables compromise 3-year lags. *** p < 0.01, ** p < 0.05, * p < 0.1.

In all the considered subsamples, results concerning the main control variables in our specification, i.e. lagged employment, turnover, gross investments, and labour cost per employee, are in line with the baseline model.

When distinguishing companies by sectoral belonging, the coefficients for AI and non-AI patents are significant among service sector firms, but not among the manufacturing firms (we recall that more than 43% of the companies in the sample are in services, about three-quarters of them in what could be considered as knowledge-intensive sectors).^21^ Compared with the baseline estimations, the positive employment impact for firms in service industries is stronger in magnitude (ranging from 2.7% to 4.5%) and applies for AI and non-AI patents defined according to both patent measures. In particular, among services, the coefficients of both AI and non-AI patent family size reach customary levels of statistical significance (95%).

Recognizing that many of today's AI innovations go back to the boom period of the ICT revolution as of the 1990s, we further investigated heterogeneous effects for sub-samples comprising firms established before and after 1990.^22^ The results for the focal patent variables show that AI and non-AI patents are only significant for the younger companies (around 2/3 of the firms in the sample). The effects observed for these firms turn out to be highly significant for both AI and non-AI patent counts, while patent family size leads to significant coefficients only for AI technologies, similar to the baseline scenario.

Some companies stand out even among the technologically active firms constituting our sample, based on the relative importance of their AI patents.^23^ The estimation results show that AI and non-AI patents are only significant for the set of AI-specialized companies. This is an important result, hinting at the magnitude of potential job creation by the leading front-runner product innovators in AI; moreover, the positive and significant effect of their non-AI patents on labour demand underlines the fact that, even for AI-specialized firms, AI turns out to be complementary to other labour-friendly innovations.

Finally, looking at the more reliable weighted estimations, in the baseline scenario and in the split regressions reported in Table 3, the labour-friendly impact of AI technologies turns out to be larger in magnitude (and sometimes more significant) than the labour-friendly effect of non-AI innovations. If we take into account jointly this outcome and the observed role of AI-intensive firms in driving the detected job-creating effects (see above), we may conclude that the emerging AI technologies are those that drive the overall positive employment impact of product innovation in the investigated companies.

## 6 Conclusions

Most previous studies have dealt only with the possible labour-saving effect of automation and robotization, which amounts to process innovations in the adopting industries. In contrast, this paper assesses the possible labour-friendly nature of AI technologies, seen as product innovations in the supply (upstream) sectors; in doing so, it feeds new evidence into the debate on the employment impact of AI technologies. Our main results can be summarised as follows.

Our overall estimates reveal a positive and significant impact of AI patent families on employment (with the estimated elasticity being about 2%), supporting the idea that product innovation in the AI supply industries is labour-friendly. Interestingly enough, this positive employment impact is additional to the job creation effect of other patenting activities. Moreover, we find that the positive employment impact is limited to service sectors and younger firms, which are the leading players in the AI revolution. Finally, some evidence of increasing returns seems to emerge; indeed, the innovative companies which are more focused on AI technologies are those obtaining greater effects in terms of job creation.

Putting together our results, we can conclude that the possible employment benefits of AI technologies, at least so far, mainly come from companies that are at the core of the current technological revolution.

The evidence suggests that technological leaders within the emerging AI paradigm can realize (moderate) labour-friendly outcomes; however, heterogeneity is also detected, with manufacturing, older and less innovative companies unable to couple product innovation with job creation.

In terms of (tentative) policy implications, these findings call for caution when considering the real magnitude of the job creation effect of new technologies: compared with the labour-saving effect of the adoption of AI technologies (massive according to some studies, see Section 2), the labour-friendly effect in the supply industries appears limited in magnitude and scope (for example, the hiring of data scientists in upstream services and AI big-7 companies would hardly compensate job losses due to robots in downstream manufacturing). Nevertheless, industrial and innovation policies should consider promoting these new and emerging sectors, being sure to achieve positive complementary targets in terms of employment creation. In particular, the revealed evidence of possible increasing returns might support an increase in subsidies aimed at those companies already fully engaged in AI patenting. At the same time, safety nets and active labour market policies continue to be necessary in order to deal with employment displacement due to the widespread diffusion of AI technologies in the user industries.

Obviously, this study is subject to some limitations that might be overcome by future research. In particular, our results are micro-based and focus on companies active in AI patenting, as discussed in Section 3. This means that our findings should not be generalized to the level of the entire economy, but are instead specific to the sub-population of companies engaged in the development of AI technologies. Moreover, although based on firm-level data, our econometric analysis is obviously unable to zoom in on and further investigate the employment decisions taken within a given company and their interactions with output, investment and innovative strategies; this unexplored perspective opens the way to further research based on in-depth case studies and dedicated surveys.

## CRediT authorship contribution statement

**G. Damioli:** Data curation, Writing – original draft. **V. Van Roy:** Data curation, Writing – original draft. **D. Vertesy:** Data curation, Writing – original draft. **M. Vivarelli:** Conceptualization, Writing – original draft.

## Declaration of competing interest

None.

## Figures and Tables

**Table A1:** List of keywords related to Artificial Intelligence.

Artificial intelligence	Evolutionary Computation	Probabilistic modeling
Artificial intelligent	Face recognition	Random Forest
Artificial reality	Facial recognition	Reinforcement learning
Augmented realities	Gesture recognition	Robot*
Augmented reality	Holographic display	Self driv*
Automatic classification	Humanoid robot	Sentiment analysis
Automatic control	Internet of things	Smart glasses
Autonomous car	Knowledge Representation	Speech Recognition
Autonomous vehicle	Machine intelligence	Statistical Learning
Bayesian modelling	Machine learn	Supervised learning
Big data	Machine to machine	Transfer Learning
Computational neuroscience	Mixed reality	Unmanned Aerial Vehicle
Computer Vision	Natural Language Processing	Unmanned aircraft system
Data mining	Neural Network	Unsupervised learning
Data science	Neuro-Linguistic Programming	Virtual reality
Decision tree	Object detection	Voice recognition
Deep learn	Predictive modelling	

**Table B1:** Description of the variables.

Variable name	Variable definition
Employment	Natural logarithm of the number of employees expressed in head counts
Turnover	Natural logarithm of the turnover expressed in EURs
Gross investments	Natural logarithm of fixed assets expressed in EURs in t - natural logarithm of fixed assets expressed in EURs in t-1
Labour cost per employee	Natural logarithm of labour cost per employee (labour cost expressed in EURs/number of employees)
AI patent families	Natural logarithm of the number of AI patent families
Non-AI patent families	Natural logarithm of the number of non-AI patent families
AI patent family size	Natural logarithm of the average size of AI patent families
Non-AI patent family size	Natural logarithm of the average size of non-AI patent families

**Table B2:** Distribution of firms across industries.

	Observations		Firms	
	Numbers	Perc.	Numbers	Perc.
Manufacturing	17,127	59.4	2,374	56.7
Primary	260	0.9	34	0.8
Food	70	0.2	13	0.3
Textile	130	0.5	20	0.5
Paper	135	0.5	18	0.4
Chemistry	896	3.1	113	2.7
Pharmaceutical	385	1.3	42	1.0
Minerals	171	0.6	24	0.6
Metal	1,263	4.4	171	4.1
Electronics	6,337	22.0	944	22.6
Machinery	4,672	16.2	627	15.0
Transport	1,712	5.9	214	5.1
Other Manufacturing	1,096	3.8	154	3.7
Services	11,713	40.6	1,810	43.3
Construction	1,001	3.5	159	3.8
Electricity/Water	187	0.7	26	0.6
Retail trade	1,827	6.3	274	6.6
Transport Services	113	0.4	19	0.5
Hotel & Catering	38	0.1	7	0.2
Telecommunication	4,641	16.1	721	17.2
Finance	144	0.5	21	0.5
Real Estate & Rental	105	0.4	22	0.5
Scientific	2,698	9.4	402	9.6
Administration/Education	794	2.8	127	3.0
Other services	165	0.6	32	0.8
Total	28,840	100.0	4,184	100.0

**Table B3:** Distribution of firms across regions and countries.

	Observations		Firms	
	Numbers	Perc.	Numbers	Perc.
Asia	16,409	56.9	2,560	61.2
South Korea	11,453	39.7	1,729	41.3
Japan	3,070	10.6	564	13.5
Taiwan	1,275	4.4	171	4.1
China	402	1.4	66	1.6
Rest of Asia	209	0.7	30	0.7
Europe	11,038	38.3	1,331	31.8
Germany	2,496	8.7	329	7.9
France	2,063	7.2	224	5.4
United Kingdom	1,593	5.5	161	3.8
Italy	1,397	4.8	162	3.9
Spain	784	2.7	98	2.3
Sweden	561	1.9	66	1.6
Rest of Europe	2,144	7.4	291	7.0
United States	1,023	3.5	239	5.7
Rest of world	370	1.3	54	1.3
Total	28,840	100.0	4,184	100.0

**Table C1:** Model selection.

	Pooled OLS	Fixed effects	SYS-GMM (one-step)
Employment t-1	0.824***	0.434***	0.447***
	(0.000)	(0.000)	(0.000)
Turnover	0.136***	0.281***	0.368***
	(0.000)	(0.000)	(0.000)
Gross investments	0.129***	0.064***	0.033*
	(0.000)	(0.000)	(0.092)
Labour cost per employee	−0.106***	−0.248***	−0.475***
	(0.000)	(0.000)	(0.000)
AI patent families	−0.001	0.017***	0.023**
	(0.928)	(0.002)	(0.042)
Non-AI patent families	0.017***	0.030***	0.022***
	(0.000)	(0.000)	(0.010)
Constant	−0.773***	0.573***	0.813
	(0.000)	(0.009)	(0.243)
R-squared	0.986	0.642	
F		224.74***	
		(0.000)	
Wald test			6,944***
			(0.000)
Hansen test			147,971***
			(0.000)
AR (1)			−12.02***
			(0.000)
AR (2)			−2.134**
			(0.033)
AR (3)			−0.634
			(0.526)
Instruments			108
Obs.	28,840	28,840	28,840
N. of firms	4,184	4,184	4,184

*Notes*: All variables are taken in natural logs, apart from gross investments, which are expressed as the log difference of fixed assets between time *t* and *t-1*. All models include year dummies. Pooled OLS and SYS-GMM models also include industry and country dummies. p-values derived from robust standard errors are reported in parentheses. For GMM estimation, instrumental variables compromise 3-year lags. *** p < 0.01, ** p < 0.05, * p < 0.1.

**Fig. C1:**
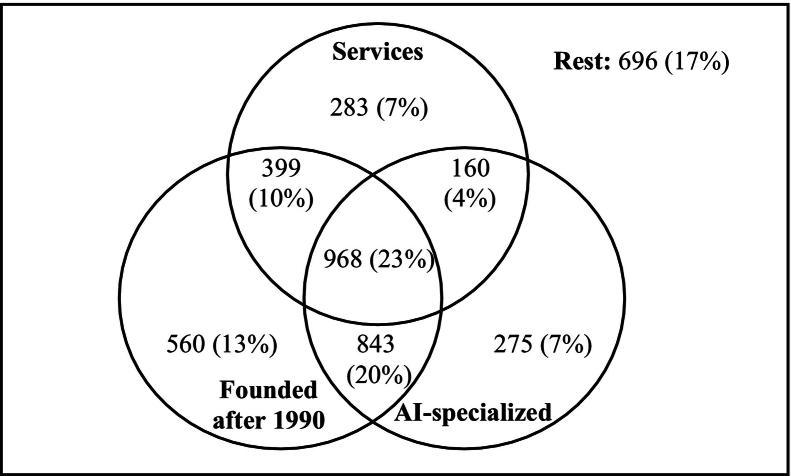
Distribution of firms across sub-samples.

## Data Availability

The authors do not have permission to share data.

## Appendix A Data collection and data cleaning

### A.1 Data collection

Fig. A1 presents a synoptic chart illustrating the data collection process. Tools for Innovation Monitoring (TIM), which is an analytics tool developed by the Joint Research Centre to support policy-making in the field of innovation and technological development, was used for the patent search. TIM provides access to patent documents in the PATSTAT database from the European Patent Office and allows for text-mining searches. TIM contains patents from more than 90 patent authorities including all the major patenting countries, and regroups patent documents per patent family when at least one of the members of the family is in English. To retrieve AI patents, text-mining techniques were used to detect the keywords present in Table A1 in either the patent title or the abstract.

Once the relevant AI patent families had been retrieved with this text-mining technique, the patent family numbers were linked with the ORBIS Intellectual Property database. Subsequently, patent applicants were linked to their BVD ID number to obtain account information on turnover, employment, fixed capital and sector. In a final step, the complete patent portfolio of the identified AI-active companies was retrieved to populate the dataset on non-AI patent families.

### A.2 Data cleaning

Data cleaning was carried out in 3 steps. The first step was the identification of the most common clerical errors or typos in the key economic variables (number of employees, turnover, fixed assets and cost of employees) from the Orbis database (for instance, missing 000s). Moreover, we used the imputation procedure put forward by Hallak and Harasztosi (2019); in particular, data for number of employees missing in a year between two known time points (not more than 4 years apart) were imputed by applying a linear interpolation. No imputation was carried out for missing gross investments and cost of employees' figures due to high annual fluctuation. Isolated unreliable zero values for turnover and −100% investment values were imputed to 10 thousand euros for turnover and −98% for investment.

In a second step, outliers in year-on-year growth rates for the key economic variables were removed (we decided to use growth rates in order to take into account a firm's size, i.e. an additional increase of 10 employees may imply a doubling in the size of a small firm, and only growing by a few percentage points for a large corporation). The thresholds applied corresponded to trimming slightly less than the bottom and top 1% of the growth distributions of the different variables.

Finally, in a third step, the top 1 percentile in levels was also trimmed with regard to both the key economic variables and the patent variables.

## Appendix C Model selection and splits

*Notes*: N = 4,184.

## Footnotes

1 The macroeconomic context is beyond the scope of the present study, but it is obvious that radical product innovation contributes significantly to economic growth, structural change and employment evolution both in the developed (see Freeman and Soete, 1994; Aghion and Howitt, 1998) and developing countries (see Hidalgo et al., 2007; Vu and Asongu, 2020).

2 For both a theoretical setting and an empirical test of an upstream/downstream model representing the sectoral patterns of job creation and job destruction, see Dosi et al. (2021).

3 In Damioli et al. (2022) we provide a concise summary of our research framework, and through a stepwise empirical analysis we distill the baseline specification which is the starting point of the present study (for a detailed summary of our previous study, see Section 2).

4 Differently from previous studies, our dataset is worldwide and in contrast to that used in Damioli et al. (2022), it builds on a broader set of keywords and updated algorithms and consequently on a larger number of observations (see next section for further details).

5 The relationship between innovation and employment is a ‘classical’ controversy, involving a clash between two views. One states that labour-saving innovations create technological unemployment, as a direct effect. The other view argues that product innovations and indirect (income and price) effects can counterbalance the direct effect of job destruction brought about by the process innovations incorporated in new machineries and equipment (for fully articulated surveys, see Pianta, 2005; Vivarelli, 2014; Calvino and Virgillito, 2018; Ugur et al., 2018; Barbieri et al., 2020; Corrocher et al., 2023; for recent studies on the employment, skill and wage impact of new technologies see Dosi et al., 2021; Goel and Nelson, 2022; Montobbio et al., 2022, Montobbio et al., 2024; Vannutelli et al., 2022).

6 Following the approach adopted by Acemoglu and Restrepo, Chiacchio et al. (2018) apply it in the context of EU labour markets. They assess the impact of industrial robots on employment and wages in 116 NUTS regions of six EU countries, namely Finland, France, Germany, Italy, Spain, and Sweden, largely representative of European automation. Their results suggest that robot introduction is negatively associated with the employment rate (one more robot per thousand workers reduces the employment/population ratio by about 0.16/0.20 %).

7 Such as the so-called “Big Seven”: Amazon, Google, Microsoft, Facebook, Alibaba, Baidu, Tencent.

8 Ours is a global dataset, differently from Alderucci et al. (2020) and Yang (2022).

9 For instance, a pooled ordinary least squares (POLS) estimation of the labour demand leads to a biased coefficient estimate of the lagged dependent variable, as the firm-specific part of the error term is positively correlated with the lagged dependent variable.

10 While this procedure solves the endogeneity issue from an econometric point of view, some concerns might remain in terms of theoretical setting and interpretation of results. Indeed, although system GMM controls for firm fixed effects through the lagged dependent variable, it is still conceivable that output, investment, innovation and employment decisions at the level of the single firm might be taken simultaneously, with a co-evolving interdependency which is difficult to grasp fully through an econometric specification and better analysed through in-depth case studies.

11 Reported in the relevant tables.

12 Recent studies (e.g. Baruffaldi et al., 2020) have also developed machine learning techniques to parse the corpus of publications and patents in order to identify AI-related content. However, such an approach is considered still at an initial stage of development, requiring further improvements in order to be able to provide robust results (Baruffaldi et al., 2020).

13 Data cleaning and outlier treatment are described in Appendix A.

14 The description of the variables used in the empirical analysis is summarised in Table B1 of Appendix B.

15 We also ran other estimations using additional splits (based, for instance, on a firm's size and on a more granular sectoral belonging), but we did not obtain relevant results (results available from the authors upon request).

16 We also tried alternative thresholds for defining a firm as AI-specialized, namely as having a ratio of above 10% or above the sample mean (15.3%), and found results analogous to those reported in the following tables.

17 The low share of firms based in the United States and China are due to a recognised bias in ORBIS, its low coverage of these countries. Given this limitation in our data, we nevertheless opted to include U.S. and China to enhance the geographic coverage of the sample. We refer to Table B3 in Appendix B for a more detailed distribution of firms across regions and countries.

18 Results are available from the authors upon request.

19 These outcomes might be due to the fact that capital formation also incorporates the adoption of process innovations.

20 As shown in the Venn diagram in Fig. C1 in the Appendix, about 83 % of the companies from the full sample are included in the union of the three groups (services, established after 1990 and AI-intensive), and about 25 % of the firms are in the intersection of the three.

21 The lack of significance of any of the patent variables in manufacturing companies is also observed when further distinguishing high-tech and low-tech manufacturing sectors (results available from the authors upon request).

22 1990 may be an arbitrary threshold; we note however that adjusting it by 5 years has limited impact on the findings (results available from the authors upon request).

23 Recall that we refer to those specializing in AI as the firms that are above the median in the relative share of AI technologies among their patent portfolios.
